# Low-Fat Salad Dressing as a Potential Probiotic Food Carrier Enriched by Cold-Pressed Tomato Seed Oil By-Product: Rheological Properties, Emulsion Stability, and Oxidative Stability

**DOI:** 10.1021/acsomega.2c06874

**Published:** 2022-12-14

**Authors:** Alican Akcicek, Rusen Metin Yildirim, Zeynep Hazal Tekin-Cakmak, Salih Karasu

**Affiliations:** † Department of Gastronomy and Culinary Arts, Faculty of Tourism, Kocaeli University, Kartepe, Kocaeli41080, Turkey; ‡ Department of Food Engineering, Faculty of Chemical and Metallurgical Engineering, Yıldız Technical University, İstanbul34220, Turkey; § Department of Nutrition and Dietetics, Health Sciences Faculty, Istinye University, İstanbul34010, Turkey

## Abstract

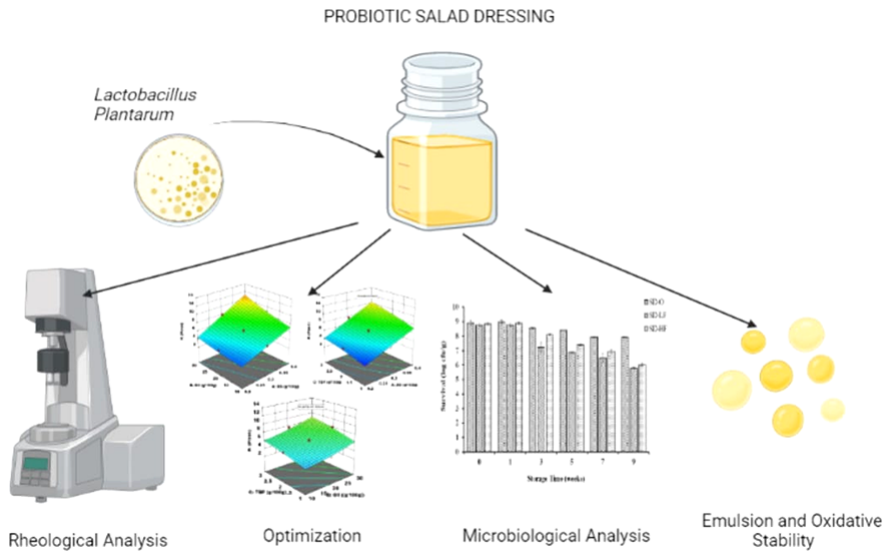

This study aims to investigate the potential of the use of cold-pressed tomato seed oil by-products in a low-fat salad dressing as potential probiotic food carriers to improve the oxidative stability and emulsion stability as well as the rheological properties. The low-fat salad dressing emulsions were formulated with cold-pressed tomato seed by-product (TBP) and *Lactobacillus plantarum* ELB90. The optimum low-fat salad dressing formulations found were determined as 10 g/100 g oil, 0.283 g/100 g xanthan, and 2.925 g/100 g TBP. The samples prepared with the optimum formulation (SD-O) were compared with the low-fat control salad dressing sample (SD-LF) and the high-fat control salad dressing sample (SD-HF) based on the rheological properties, emulsion stability, oxidative stability, and *L. plantarum* ELB90 viability. The sample SD-O showed shear-thinning, viscoelastic solid, and recoverable characters. The sample SD-O showed higher IP and Δ*G*^++^ and lower Δ*S*^++^ values than those of the control samples. After 9 weeks of refrigerated storage, viable *L. plantarum* ELB90 cell counts of salad dressing samples were counted as 7.93 ± 0.03, 5.81 ± 0.04, and 6.02 ± 0.08 log cfu g^–1^ for SD-O, SD-LF, and SD-HF, respectively. This study showed that TBP could be successfully used in a low-fat salad dressing as a potential probiotic carrier.

## 1 Introduction

Salad dressing is an oil-in-water emulsion product containing around 30% oil. Fat consumption is associated with some health problems such as obesity and cardiovascular diseases.^1^ Therefore, in recent years, there has been an increasing trend toward the consumption of reduced-fat products.^2^ Oil is one of the main components determining the texture, physical stability, and organoleptic properties of emulsions like salad dressing. Finding new texture enhancers or fat substitutes to compensate for the reduction in textural and sensory qualities with the reduction of fat is the most important challenge for low-fat emulsions.^2^ Therefore, studies on alternative fat substitutes should be conducted.

Tomato seeds are the major by-products of the tomato paste manufacturing industry and contain 3.3% ash, 17.3% oil, and 27.2% protein.^3^ Tomato seed oil (TSO) is considered a good source of edible oil and one of the major food ingredients across the world. While obtaining cold-pressed oils, they are not subjected to chemical applications such as high heat treatment and refining and solvent extraction, so the nutritional components and sensory properties of these oils change much less than refined oils.^4^ After cold pressing, oil and water are removed and by-products with highly nutritious components are formed, predominantly protein and carbohydrate.^5^ Therefore, cold-pressed by-products could also be used as fat substitutes in low-fat O/W emulsions such as low-fat salad dressing or mayonnaise.^6−9^

Consumer desire for healthier meals has grown significantly over the past several decades, which served as the catalyst for the creation of functional food products by including components having one or more positive impacts on human health. The basic aim of functional foods is to maintain intestinal health. Due to their potential to improve the composition and activity of the gut’s microbial population and general health, probiotics and/or prebiotics have attracted attention as dietary supplements.^10^

The majority of probiotics are now commercially available in fermented dairy products; however, research is being conducted to create probiotics that may be consumed without dairy due to lactose intolerance, allergies to milk proteins, cholesterol, and saturated fatty acid levels.^11^ When considering nondairy alternatives as potential probiotic carriers, cold-pressed oil by-products have a number of benefits, including being rich sources of proteins, carbs, and bioactive substances.

Mantzouridou et al.^12^ suggested that probiotic bacteria can be effectively protected and have their survival rates increased in simulated gastrointestinal tract settings by being entrapped in emulsion droplets or in the inner-water phase of water/oil/water (W/O/W) emulsions. It was reported that the formation of an inulin-based dressing emulsion as a potential probiotic food carrier and entrapment of the probiotic *Lactobacillus paracasei* subsp. paracasei DC412 in the oil phase of protein-stabilized emulsions protected the cells when exposed to GI tract enzymes, provided the emulsions were freshly prepared. However, after treatment of aged emulsions for up to 4 weeks under conditions simulating the human GI environment, the microorganism could not survive in sufficient quantities. Mantzouridou, Spanou, and Kiosseoglou^13^ investigated the formulation optimization of a potential prebiotic low-in-oil oat-based salad dressing for increasing *Lactobacillus paracasei* subsp. paracasei survivability. They reported that after 7.5 weeks of refrigerated storage and treatment with simulated gastric and intestinal liquids, viable cell counts in emulsions reached 10^8^ cfu g^–1^.

This study investigated whether the salad dressing sample containing tomato seed oil by-product (TBP) could act as a probiotic food carrier to maintain the viability of *Lactobacillus plantarum* ELB90 cells during storage. In this study, optimization was made to determine the optimum composition contents for low-fat salad dressing containing TBP. In addition, the effect of low- and high-fat salad dressing samples and a determined optimum low-fat salad dressing on the rheological properties and oxidative stability and emulsion stability were investigated.

## 2 Material and Methods

### 2.1 Material

Tomato seed oil and by-product (TBP) was obtained from Tazemiz Foods Company (Manisa, Turkey). TBP was sieved through mesh no. 140. After grinding of by-products, they were stored in a closed light-free polypropylene bag at 10 °C until their analysis. Other ingredients used in salad dressing formulations, namely, sunflower oil, vinegar, and salt, were obtained from a local market. Xanthan gum (XG) and lecithin were supplied by Sigma-Aldrich (Sigma Chemical Co., St. Louis, Missouri). After being taken to the laboratory, the products were stored at 25 °C in a polyethylene bag.

### 2.2 Method

#### 2.2.1 Characterization of TBP

The moisture, ash, protein, and lipids were determined according to official methods.^14^ The results were expressed as g/100 g. Total phenolic content was determined by means of the Folin–Ciocalteu reagent,^15^ and the total antioxidant capacity was determined by means of DPPH and ABTS radicals.^16^

#### 2.2.2 Salad Dressing Preparation

The salad dressing samples were prepared according to procedures described by Tekin and Karasu.^17^ When salad dressing samples were prepared, XG (0.35%) was first dispersed at 25 °C in water. Then, the dispersion was heated to 80 °C and agitated for 20 min, followed by the addition of a by-product. The dispersion was cooled to 25 °C after salt was dissolved in it. After dissolving the materials, the XG was hydrated by stirring at 1000 rpm in a magnetic stirrer for 6 h. The obtained dispersion was combined with sunflower oil and lecithin (3%) and homogenized for 3 min utilizing the Ultra Turrax (Daihan, HG15D) at 10,000 rpm. Finally, the salad dressing was obtained and pasteurized for 10 min at 65 °C. Salad dressing samples were poured into brown bottles after homogenization and stored at 25 °C. All materials used in this experiment (beakers, brown tubes, and probes) were sterilized for 15 min at 121 °C.^12^

#### 2.2.3 Experimental Design

Response surface methodology (RSM) and full factorial central composite design (CCD) were performed to determine the optimum content of XG (%), oil (%), and TSB (%) to prepare the low-fat salad dressing. As exhibited in Table 1, 17 different experimental points were obtained using Design Expert Software (Version 7; Stat-Easy Co., Minneapolis, MN) to find the optimum conditions. For the estimation of the error, the design comprised three of the factorial points. The rheological properties of commercially produced salad dressing were taken into consideration in the selection of TBP, XG, and oil ratio. In our previous study,^9^*K* values of commercially produced salad dressings were determined. These values were taken into consideration in the selection of the TBP, XG, and oil ratio corresponding to *K* values. The *K*, *K*′, and *K*″ are response variables, and TBP, XG, and oil are process factors. A quadratic model was fitted to the experimental data for each response. Model applicability was determined based on the *R*^2^, *R*^2^-adj, lack of fit, *F*, and *p*-values obtained from ANOVA. The optimization was performed based on the highest desirability value. The formulation, including the lowest oil content with a desirability value of 1, was chosen as the optimum formulation. Three central points were used. Analysis of all points was conducted in triplicate, and the results were reported as mean values and standard deviations.

**Table 1:** Composition of TBP^a^

	TBP (%)
carbohydrate (CHO)	39.31
protein	40.08
oil	10.92
moisture	5.94
ash	3.75

a TBP: cold-pressed tomato seed oil.

Flow behavior and dynamic rheological properties of 17 different experimental points will be determined. Then, the flow behavior, dynamic rheological properties and 3-ITT rheological properties, emulsion stability, oxidative stability, and prebiotic activity will be performed on the optimum salad dressing sample (SD-O), the high-fat control sample (SD-HF), and the low-fat control sample (SD-LF).

#### 2.2.4 Rheological Analyses

A temperature-controlled rheometer (MCR 302; Anton Paar, Sydney, NSW, Austria) was used to evaluate the rheological analyses, namely, flow behavior properties, dynamic rheology, and 3-ITT rheological properties, as well as emulsion stability (thermal loop test) of salad dressings at 25 °C.

The flow behavior rheological properties of the salad dressings were measured by utilizing a parallel-plate configuration and a distance of 0.5 mm between the rheometer probe and the sample plate in the range of 0–100 shear rate (s^–1^). A sample of weight 2 g was added to hold the rheometer measurement plate until the temperature was achieved; then, analysis was performed. The shear stress values corresponding to the shear rate were measured. The power-law model and nonlinear regression were used to evaluate the parameters of the flow behavior and rheological properties.
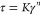
1

In eq 1, τ shows the shear stress (Pa), *K* is the consistency index (Pa·s^–n^), γ is the shear rate (s^–1^), and *n* is the flow behavior index.

A parallel-plate configuration was used to conduct dynamic rheological analysis of the samples. To evaluate the linear viscoelastic region (LVR), the amplitude sweep test was conducted first with a strain value of 0.1%. In LVR, the frequency sweep test was performed in the 0.1–10 Hz and 0.1–64 (ω) angular speed ranges. In addition to angular velocity and frequency, the storage modulus (*G*′) and loss modulus (*G*″) were calculated. The power-law model and nonlinear regression were used to assess parameters specific to complex rheological properties.^18^
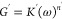
2
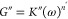
3

In eqs 2 and 3, *G*′ (Pa) is the storage modulus, *G*″ (Pa) is the loss modulus, ω is the angular velocity value (s^–1^), and *K*′, *K*″, and consistency index values *n*′ represent the flow behavior index values.

For the salad dressing samples, the 3-ITT rheological properties were estimated to be constant shear rate 0.5 s^–1^ and variable shear rate 150 s^–1^. LVR of the samples was taken into account as the values were chosen, and the LVR of the samples ends at 50 s^–1^. In the first time interval, salad dressing samples were exposed for 100 s at a very low shear rate (0.5 s^–1^). In the second time interval, 150 s^–1^ was subjected to the specified shear force for 40 s. In the third time interval, the dynamic rheological behavior in the second time interval was tested by exposing the samples to a low shear rate in the first time interval. It was observed that there was a change in the viscoelastic solid structure (*G*) of the salad dressing samples. The behavior of the salad dressing samples in the third time interval was modeled using a second-order structural kinetic model (*n* = 2)
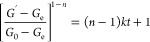
4

In eq 4, *G*′ represents the storage module in (Pa), *k* is the thixotropic rate constant, *G*_0_ is an initial storage modal value (Pa) in the third time interval, and *G*_e_ is the equilibrium storage modulus (product completely recovers itself).^19^

#### 2.2.5 Emulsion Stability

The thermal loop test was used to determine the physical stability of the oil-in-water emulsions subjected to 11 thermal cycles under high temperatures (from 23 to 45 °C) and low temperatures (from 5 to 23 °C).^20^ A thermal loop test is an appropriate method for evaluating the stability of the emulsion in a short time by determining structural changes and simulating temperature differences in manufacturing, distribution, storage, and transport. The emulsions are subjected to different numbers of thermal cycles. The change of modules from cycle to cycle expresses the structural or morphological changes caused by applying thermal stress. The thermal loop test can be used as a quick tool for predicting oil stability in water emulsions such as salad and mayonnaise.

The strain value and angular frequency (ω) were arranged as 0.5% and 10 Hz, respectively. The cooling and heating rates were set to 11 K min^–1^. Using the internal loop, the maximum points of all cycles were determined with rheometer software. The relative structural change value (Δ) was calculated for G* values by dividing the maximum value of each cycle by the value of the first cycle using eq 5 to observe the thermal stability of salad dressing.^21^
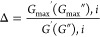
5

#### 2.2.6 Oxidative Stability

The oxidative stability of the salad dressing samples was tested using the OXITEST Device (Velp Scientifica, Usmate, MB, Italy).^22^ All samples were weighed before the oxidative stability analysis started. First, 8 g of sunflower oil was weighed into the sample cells. Then, 20 g of each salad dressing sample was weighed into the sample cells. The device temperatures and the oxygen pressure were adjusted at 80, 90, 100, and 110 °C and 6 bar, respectively. The induction period (IP) value obtained by the OXITEST system was used to interpret the oxidative stability values of the samples.

#### 2.2.7 Preparation of Lactic Acid Bacteria for Inoculation

The lactic acid bacterium used in this study was *L. plantarum* ELB90.^23^ It was stored at −80 °C in MRS broth with 25% glycerol. Prior to use for salad dressing production, the strain was grown overnight in 50 mL of MRS broth at 37 °C. The grown culture of *L. plantarum* ELB90 was centrifuged (6000*g* for 10 min at 4 °C) and washed twice with sterile distilled water and inoculated into the salad dressing samples to get an initial cell count of 10^9^ mL^–1^.

#### 2.2.8 Enumeration of *L. plantarum* ELB90

To determine the enumeration of LAB, 10 g of salad dressing was homogenized with 90 mL of sterile peptone water and serially diluted. MRS agar (Merck, Germany) was used to determine LAB contents. It was incubated at 37 °C for 48 h and counted.

#### 2.2.9 Statistical Analysis

The results of the rheological analyses were calculated using the power-law model parameters by nonlinear regression analysis. The applicability of the model was evaluated by the coefficient of determination (*R*^2^). The nonlinear regression analyses were performed using the Statistica software program (StatSoft, Inc., Tulsa, OK). For the comparison of the power-law parameters, means and standard deviations were calculated with SPSS statistical software (Version 16.0 SPSS Inc., Chicago, Illinois). Duncan’s multiple-comparison tests were used in the 0.05 confidence interval.

## 3 Results and Discussion

### 3.1 Characterization of TBP

The carbohydrate (CHO), protein, oil, moisture, and ash content of TBP were determined as 39.31, 40.08, 10.92, 5.94, and 3.75%, respectively. Karaman, Karasu, Tornuk, Toker, Geçgel, Sagdic, Ozcan, and Gül^24^ reported that oil, protein, and crude fiber contents of the cold-pressed almond, walnut, pomegranate, and grape by-products were 4.82–12.57, 9.38–49.05, and 5.87–45.83%, respectively. Also, Tekin-Cakmak, Karasu, Kayacan-Cakmakoglu, and Akman^6^ investigated the physicochemical properties of by-products (black cumin seed, coconut, flaxseed, and pumpkin seed) from the cold-pressed oil industry. They reported that protein, oil, and CHO contents of these by-products were 16.36–44.02, 9.04–19.38, and 32.26–60.82%, respectively. When all of these results are examined, it can be concluded that cold-pressed oil industry wastes are rich in protein and CHO.

TPC, DPPH, and ABTS of TBP were determined as 115.05 ± 1.30 mg GAE/100 g TBP, 20.51 ± 0.48 mg TE/100 g TBP, and 7.29 ± 0.14 mg TE/100 g TBP, respectively. Since cold-pressed oils are extracted by the screw press method without heat and chemical treatment, cold-pressed oil by-products also have higher amounts of bioactive compounds and flavor and aroma compounds compared with other oil by-products.^25^

### 3.2 Rheological Properties

#### 3.2.1 Steady-Shear Rheological Properties

**Figure 1:**
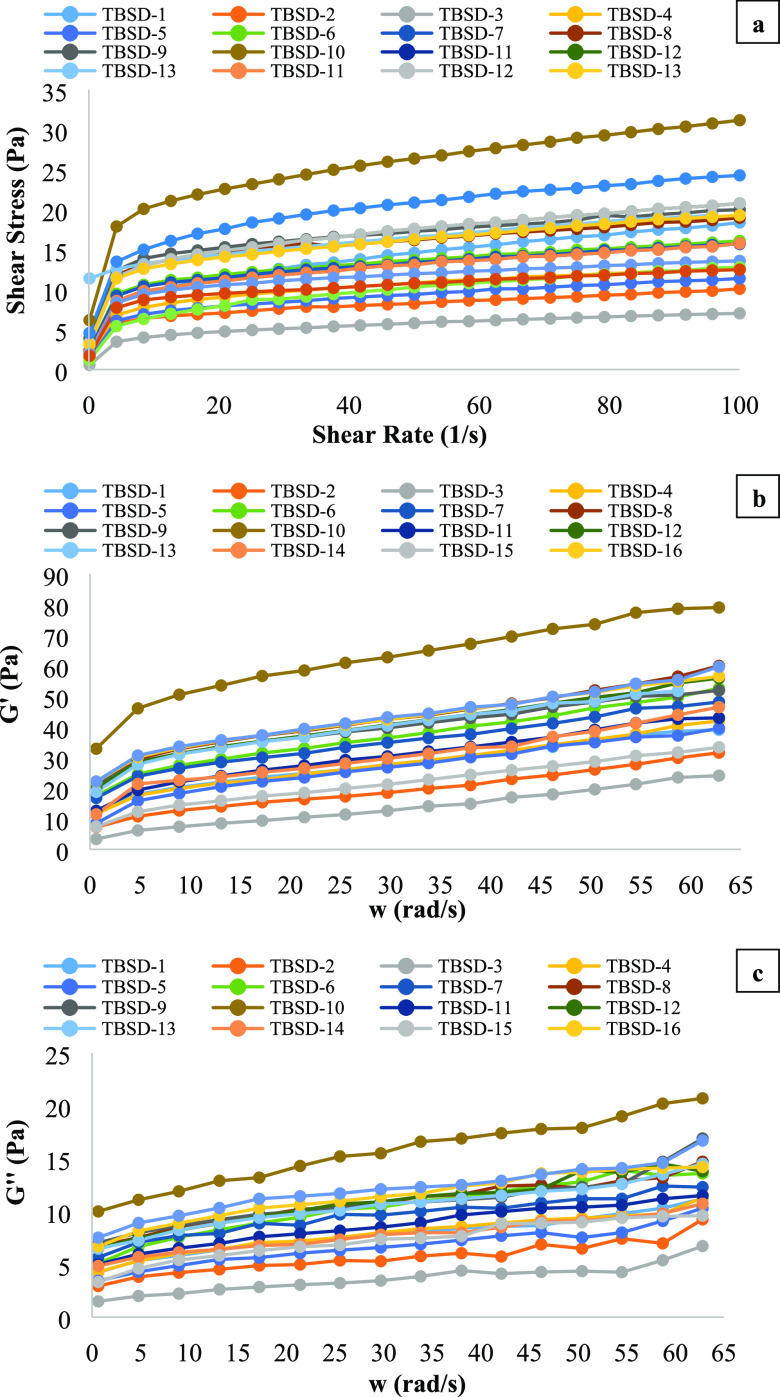
Steady and viscoelastic rheological properties of the salad dressing samples.

Figure 1a shows the flow curves (i.e., the shear stress as a function of shear rate) for salad dressing samples. As can be seen in Figure 1a, all samples showed shear-thinning flow behavior, explaining the dramatic shear-induced structural breakdown related to the mechanism of oil droplet deflocculating.^26^ This flow behavior is the typical behavior for salad dressing samples and shows that the salad dressing samples exhibit pseudoplastic flow.^27^ TBSD-10 (0.4 g/100 g XG, 30 g/100 g oil, and 3 g/100 g TBP) indicated the highest pseudoplastic property due to high fat and thickener contents.

Table 2 indicates that the data obtained from flow behavior rheological measurements of 17 different formulations were evaluated according to the power-law model. The power-law model was fitted with experimental rheological data of the samples (*R*^2^: 0.982–0.999). The flow consistency index (*K*) and flow behavior index (*n*) values of the samples were calculated using the power-law model.

**Table 2:** Flow Behavior of Salad Dressing Samples Containing TBP^a^

samples	XG (g/100 g)	oil (g/100 g)	TBP (g/100 g)	*K*(Pa·s*^n^*)	*n*	*R*^2^
TBSD-1	0.2	30	3	4.994 ± 0.032	0.280 ± 0.004	0.997
TBSD-2	0.2	10	3	3.551 ± 0.179	0.218 ± 0.008	0.9840
TBSD-3	0.2	10	1	2.155 ± 0.045	0.252 ± 0.001	0.9930
TBSD-4	0.3	20	1	4.570 ± 0.033	0.222 ± 0.001	0.9924
TBSD-5	0.2	20	2	3.959 ± 0.007	0.226 ± 0.001	0.9900
TBSD-6	0.3	20	2	6.375 ± 0.100	0.199 ± 0.004	0.9882
TBSD-7	0.3	20	2	6.230 ± 0.065	0.200 ± 0.003	0.9881
TBSD-8	0.4	10	3	8.247 ± 0.136	0.180 ± 0.006	0.9823
TBSD-9	0.4	20	2	8.734 ± 0.114	0.178 ± 0.003	0.9851
TBSD-10	0.4	30	3	12.680 ± 0.234	0.193 ± 0.003	0.9993
TBSD-11	0.3	20	2	5.648 ± 0.114	0.221 ± 0.006	0.9991
TBSD-12	0.3	30	2	7.990 ± 0.131	0.206 ± 0.002	0.9993
TBSD-13	0.3	20	3	7.658 ± 0.015	0.199 ± 0.001	0.9898
TBSD-14	0.4	10	1	5.851 ± 0.063	0.185 ± 0.003	0.9890
TBSD-15	0.2	30	1	3.383 ± 0.080	0.294 ± 0.002	0.9999
TBSD-16	0.4	30	1	9.335 ± 0.113	0.207 ± 0.002	0.9996
TBSD-17	0.3	10	2	5.228 ± 0.084	0.189 ± 0.004	0.9898

a TBSD, salad dressing samples containing TBP; *K*, consistency coefficient (Pa·s*^n^*); *n*, flow behavior index value; *R*^2^, coefficient of determination.

*K* values changed from 2.155 to 12.680 Pa s*^n^*, and *n* values were between 0.178 and 0.280. *K* and *n* values differed depending on the different formulations of salad dressings. TBSD-3 (0.2 g/100 g XG, 10 g/100 g oil, and 1 g/100 g TBP) had the lowest *K* value, while TBSD-10 (0.4 g/100 g XG, 30 g/100 g oil, and 3 g/100 g TBP) had the highest *K* value, explaining that *K* values depend on the amount of oil, XG, and by-product. Although TBSD-8 and TBSD-9 contain the same amount of XG (0.4 g/100 g), TBSD-8 has nearly the same *K* values as TBSD-9, with 50% less fat content and just 1 g more TBP. When the first three samples were examined, despite the low XG amounts (0.2 g/100 g), the *K* value changed from 4.994 to 3.551 Pa s*^n^* when the oil ratio was reduced from 30 g/100 g to 10 g/100 g and remained constant at TBP (3 g/100 g). The *K* value became 2.155 Pa s*^n^* when the TBP content decreased to 1 g/100 g along with the oil content. These results showed that the decrease in oil content can be compensated by TBP as a flow behavior characteristic. On the other hand, it has been seen that the *n* values are less than 1, the salad dressing samples show non-Newtonian properties, and the *n* values decrease with the increase in the consistency coefficient. In salad dressings, on the other hand, it is desired that the *n* value is close to zero, that is, it is pseudoplastic.

#### 3.2.2 Viscoelastic Rheological Properties

Figure 1b,c shows the dynamic rheological properties of salad dressings obtained from 17 different formulations. In Figure 1b,c, the magnitude of both the storage modulus (*G*′) and the loss modulus (*G*″) increased with frequency. Also, all samples showed viscoelastic behavior as the *G*′ of all of the samples was higher than the *G*″, indicating a gel-like structure of a flocculated and entangled network.^2^ Generally, emulsions with higher oil contents also have higher *G*′ values.^28^ In this study, TBSD-10 (0.4 g/100 g XG, 30 g/100 g oil, and 3 g/100 g TBP) had the highest *G*′ values due to the high oil content, followed by TBSD-17 (0.3 g/100 g XG, 10 g/100 g oil, and 2 g/100 g TBP). It is explained by the interaction XG and TBP instead of oil addition in the formulation, which strengthens the gel structure of salad dressing samples. In the literature, it is seen that plant-based proteins such as soybean,^29^ lupin,^30^ and wheat^31^ proteins were used as emulsifiers, and it can be concluded that TBP acts as an emulsifier with a protein content of 40.08%.

Viscoelastic parameters of salad dressing samples were calculated using the power-law model (Table 3). As can be seen in Table 3, the *K*′ and *K*″ values of the samples are in the ranges of 1.169–33.806 and 0.880–8.490 Pa s*^n^*, respectively, while the *n*′ and *n*″ values were found in the ranges of 0.207–0.708 and 0.189–0.470, respectively (*R*^2^ = 0.951–0.986 and *R*^2^ = 0.908–0.979, respectively).

**Table 3:** Power-Law Parameters Defining Dynamic Rheological Properties of the Salad Dressing Samples Containing TBP^a^

samples	XG (g/100 g)	oil (g/100 g)	TBP (g/100 g)	*K*′ (Pa s*^n^*)	*n*′	*R*^2^	*K*″ (Pa s*^n^*)	*n*″	*R*^2^
TBSD-1	0.2	30	3	9.494 ± 0.292	0.330 ± 0.006	0.975 ± 0.001	3.377 ± 0.052	0.261 ± 0.006	0.9944
TBSD-2	0.2	10	3	4.135 ± 0.093	0.470 ± 0.007	0.966 ± 0.001	2.254 ± 0.042	0.470 ± 0.007	0.9920
TBSD-3	0.2	10	1	1.169 ± 0.218	0.708 ± 0.030	0.978 ± 0.002	0.880 ± 0.063	0.412 ± 0.038	0.9908
TBSD-4	0.3	20	1	8.655 ± 0.088	0.359 ± 0.000	0.972 ± 0.001	3.611 ± 0.040	0.241 ± 0.006	0.9598
TBSD-5	0.2	20	2	6.802 ± 0.855	0.396 ± 0.012	0.979 ± 0.010	2.672 ± 0.052	0.270 ± 0.019	0.9937
TBSD-6	0.3	20	2	14.215 ± 0.040	0.305 ± 0.010	0.974 ± 0.000	4.579 ± 0.363	0.275 ± 0.005	0.9979
TBSD-7	0.3	20	2	13.553 ± 0.755	0.277 ± 0.002	0.961 ± 0.012	4.986 ± 0.117	0.189 ± 0.009	0.9948
TBSD-8	0.4	10	3	18.160 ± 0.054	0.266 ± 0.005	0.972 ± 0.001	5.616 ± 0.204	0.206 ± 0.001	0.9593
TBSD-9	0.4	20	2	18.823 ± 0.016	0.239 ± 0.004	0.971 ± 0.003	5.895 ± 0.365	0.208 ± 0.005	0.9598
TBSD-10	0.4	30	3	33.806 ± 2.093	0.207 ± 0.007	0.985 ± 0.001	8.490 ± 0.441	0.198 ± 0.009	0.9922
TBSD-11	0.3	20	2	10.277 ± 0.278	0.332 ± 0.003	0.972 ± 0.008	4.319 ± 0.298	0.213 ± 0.026	0.9957
TBSD-12	0.3	30	2	17.819 ± 0.187	0.260 ± 0.002	0.973 ± 0.000	5.147 ± 0.097	0.232 ± 0.003	0.9950
TBSD-13	0.3	20	3	17.813 ± 0.165	0.267 ± 0.011	0.986 ± 0.000	5.215 ± 0.128	0.223 ± 0.010	0.9952
TBSD-14	0.4	10	1	10.531 ± 0.618	0.342 ± 0.005	0.967 ± 0.007	3.800 ± 0.149	0.217 ± 0.006	0.9935
TBSD-15	0.2	30	1	5.172 ± 0.304	0.437 ± 0.017	0.980 ± 0.002	2.861 ± 0.011	0.280 ± 0.007	0.9984
TBSD-16	0.4	30	1	19.658 ± 0.058	0.243 ± 0.003	0.972 ± 0.04	6.248 ± 0.367	0.198 ± 0.009	0.9956
TBSD-17	0.3	10	2	9.223 ± 0.119	0.322 ± 0.002	0.951 ± 0.000	4.199 ± 0.302	0.215 ± 0.048	0.9935

a TBSD, salad dressing samples containing TBP; *K*′ and *K*″, consistency coefficient (Pa·s*^n^*); *n*′ and *n*″, flow behavior index values; *R*^2^, coefficient of determination.

The *K*′ value was found to be higher than the *K*″ value in all samples, showing that the elastic solid character is dominant over the viscous character. TBSD-10 (0.4 g/100 g XG, 30 g/100 g oil, and 3 g/100 g TBP) also had the highest *K*′ and *K*″ values. TBSD-3 (0.2 g/100 g XG, 10 g/100 g oil, and 1 g/100 g TBP) had the lowest *K*′ and *K*″ values.

#### 3.2.3 Determination of the Optimum Formulation of Salad Dressing

**Figure 2:**
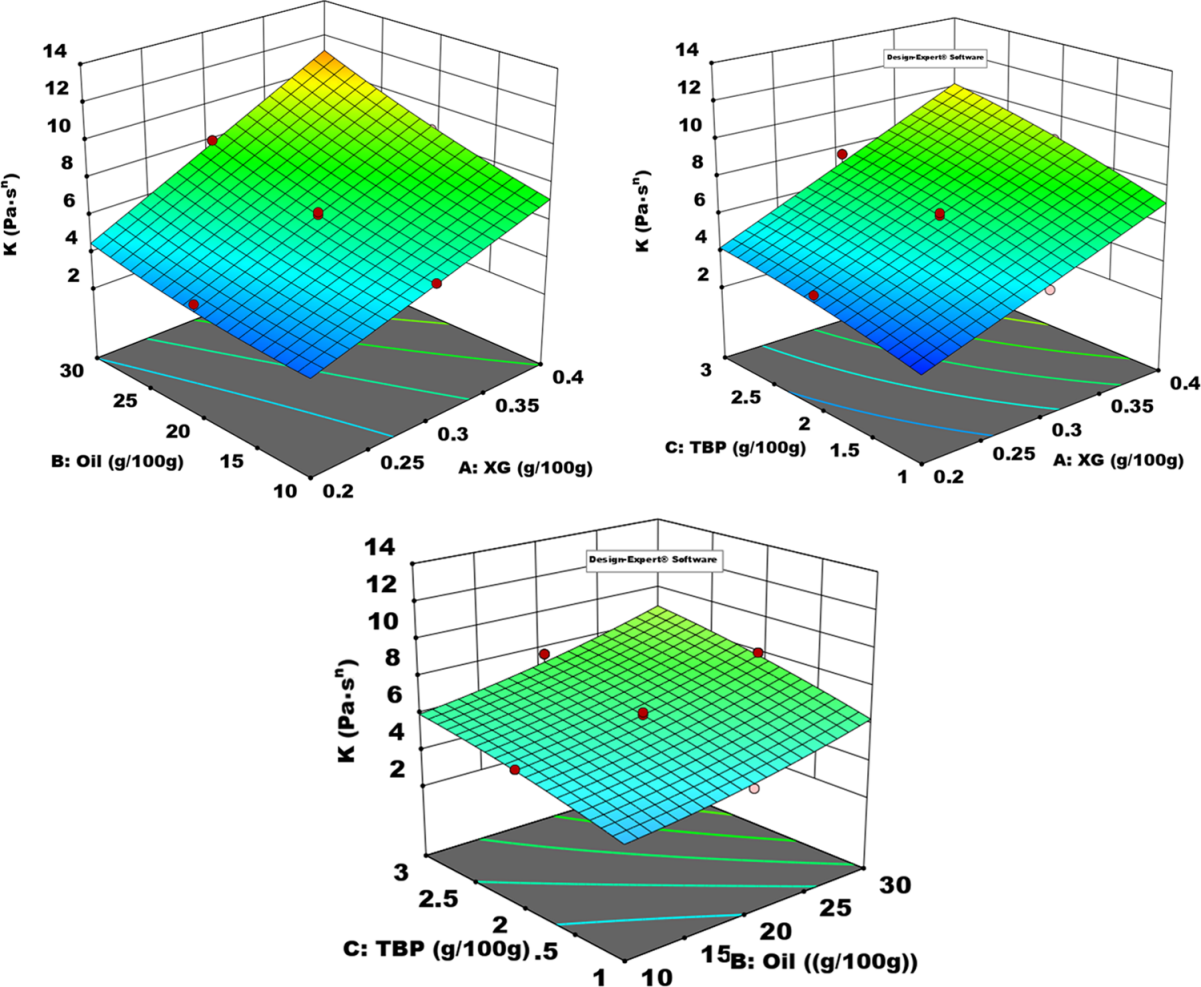
Effect of model formulation on the consistency of samples. *K*, consistency coefficient (Pa s*^n^*); TBP, cold-pressed tomato oil by-products (g/100 g); XG, xanthan gum (g/100 g).

Figure 2 shows the effect of TBP, oil, and XG contents and their interactions on the *K* value of salad dressing samples. As can be seen, a dramatic increase in the *K* value was observed as the amount of each component increased. This shows that TBP, oil, and XG in the formulation significantly affect the consistency of the salad dressing. A quadratic model was used to mathematically evaluate the effect of TBP, oil, and XG on the *K* value and to find the optimum amount depending on these three components.

Quadratic model parameters are shown in Table 4. The model *R*^2^, predicted *R*^2^, and adjusted *R*^2^ values were determined as 0.9883, 0.9654, and 0.9813, respectively. The high *R*^2^ values showed that the quadratic model could express the effect of TBP, oil, and XG on the *K* value of salad dressing quite successfully. *P*-values less than 0.0500 indicate that the model terms are significant. In this case, A, B, C, AC, and BC are significant model terms. The very low *p*-value of each ingredient (<0.0001) indicates a significant effect of these ingredients on the consistency of the salad dressing. Especially the fact that the TBP is as effective as oil and XG indicates that TBP can be a successful fat substitute in products with reduced fat.

**Table 4:** Significance of the Regression Models Established (*F* and *p*-value) as a Result of RSM

source	sum of squares	df	mean squares	*F*-value	*p*-value	
model	108.01	6	18.00	140.46	<0.0001	significant
A-TBP	13.80	1	13.80	107.65	<0.0001	
B-oil	17.82	1	17.82	139.06	<0.0001	
C-XG	71.85	1	71.85	560.62	<0.0001	
AB	0.1694	1	0.1694	1.32	0.2771	
AC	0.9343	1	0.9343	7.29	0.0223	
BC	3.44	1	3.44	26.84	0.0004	
residual	1.28	10	0.1282			
lack of fit	0.9293	8	0.1162	0.6593	0.7236	not significant
pure error	0.3524	2	0.1762			
cor total	109.30	16				
*R*^2^	0.9883					
adjusted *R*^2^	0.9812					
predicted *R*^2^	0.9654					

In this case, besides the effects of each component alone, TBP–XG and XG–oil interactions are also important. While these results increased the *K* value of TBP alone, it also increased the *K* value by interacting with XG. Thanks to the hydrophilic interactions of the fibers and proteins in TBP with XG, water molecules may be attached and an increase in *K* value may be observed. The optimum formulation was determined based on the minimum oil content.

In this study, the aim was to obtain the optimum low-fat salad dressing (SD-O) using TBP with properties similar to high-fat salad dressing. For this purpose, the minimum oil content (10%) with a desirability value of 1.00 (showing the *K* value of the full-fat control samples) was chosen as the criterion to determine the optimum formulation. The formulation of SD-O was determined as 10% oil, 0.283% XG, and 2.925% TBP. We prepared the optimum (SD-O) and control samples (HF-SD and LF-SD) and compared them to validate the experimental data and RSM results.

### 3.3 Analyses of SD-O, SD-LF, and SD-HF

#### 3.3.1 Rheological Properties of SD-O, SD-LF, and SD-HF

Table 5 indicates the steady-shear, dynamic, and thixotropic properties of SD-HF, SD-LF, and SD-O. *K* and *n* values of SD-O 5.93 Pa s*^n^* and 0.19 were determined as showing that the correlation between the experimental and predicted data was high and the response model successfully described the optimization process.

**Table 5:** Rheological Properties of Control and Optimum Salad Dressing Samples^a^

Flow Behavior Properties						
	XG (g/100 g)	oil (g/100 g)	TBP	*K*(Pa s*^n^*)	*n*	*R*^2^
SD-HF	0.40	30		6.07 ± 0.07^a^	0.20 ± 0.00	0.9999
SD-LF	0.40	10		4.16 ± 0.04^b^	0.22 ± 0.00	0.9996
SD-O	0.283	10	2.925	5.93 ± 0.13^a^	0.19 ± 0.00	0.9997

Dynamic Behavior Properties						
	*K*′ (Pa·s*^n^*)	*n*′	*R*^2^	*K*″ (Pa s*^n^*)	*n*″	*R*^2^
SD-HF	14.82 ± 0.31^a^	0.30 ± 0.00	0.9995	5.25 ± 0.02^a^	0.20 ± 0.00	0.9235
SD-LF	3.61 ± 0.02^c^	0.51 ± 0.01	0.9885	2.53 ± 0.01^b^	0.24 ± 0.00	0.9445
SD-O	10.10 ± 0.18^b^	0.34 ± 0.00	0.9775	4.94 ± 0.06^a^	0.21 ± 0.00	0.9695

3-ITT Rheological Properties						
	*G*_0_	*G*_e_	*G*_e_/*G*_0_	*k* × 1000	*R*^2^	deformation (%)
SD-HF	18.08 ± 0.12	27.68 ± 0.14	1.53 ± 0.01^a^	19.13 ± 0.21^c^	>0.99	40.12 ± 0.54^b^
SD-LF	8.44 ± 0.03	12.47 ± 0.22	1.48 ± 0.02^b^	24.01 ± 0.19^b^	>0.99	33.51 ± 0.78^c^
SD-O	10.06 ± 0.07	14.46 ± 0.09	1.58 ± 0.03^a^	36.06 ± 0.40^a^	>0.99	45.65 ± 1.46^a^

a SD-LF, low-fat control salad dressing sample; SD-HF, high-fat control salad dressing sample; SD-O, low-fat optimum salad dressing sample; *K*, *K*′, and *K*″, consistency coefficient (Pa·s*^n^*); *n*, *n*′, and *n*″, flow behavior index values; *G*_0_, initial values of the storage modulus; *G*_e_, equilibrium storage modulus; *k*, rate constant of recovery of the sample; *R*^2^, coefficient of determination. Low-fat control salad dressing samples contain 10% vegetable oil, 2% lecithin, and 0.4% XG; high-fat control salad dressing samples contain 30% vegetable oil, 2% lecithin, and 0.4% XG; and low-fat optimum salad dressing samples contain 10% vegetable oil, 2% lecithin, 0.283% XG, and 2.925% TBP. A different uppercase letter in the same column indicates statistical significance.

All samples are non-Newtonian fluids and indicate pseudoplastic behavior because of *n* < 1. Also, viscoelastic parameters of SD-O, namely, *K*′, *K*″, *n*′, and *n*″ values were 10.10 Pa s*^n^*, 4.24 Pa s*^n^*, 0.34, and 0.21, while *K*′, *K*″, *n*′, and *n*″ values of SD-HF were 14.82 Pa s*^n^*, 5.25 Pa s*^n^*, 0.30, and 0.20, respectively. The *K*′ value was found to be higher than the *K*″ value in all samples, showing that the elastic solid character is dominant over the viscous character. These values of SD-HF and SD-O samples were similar so that TBP can be used as a fat substitute for low-fat salad dressing samples. Table 5 also shows that 3-ITT parameters (*G*_0_, *G*_e_, *G*_e_/*G*_0_, *k* × 1000) were obtained with the second-order structural kinetic model. *G*_0_, *G*_e_, *G*_e_/*G*_0_, *k* × 1000, and *R*^2^ values were 8.44–18.08, 12.47–27.68, 1.48–1.58, 19.13–36.06, and >0.99, respectively. SD-O showed the highest *G*_e_/*G*_0_ (1.58) and *k* × 1000 (36.06) values, meaning that the SD-O sample showed the highest thixotropic behavior.

**Figure 3:**
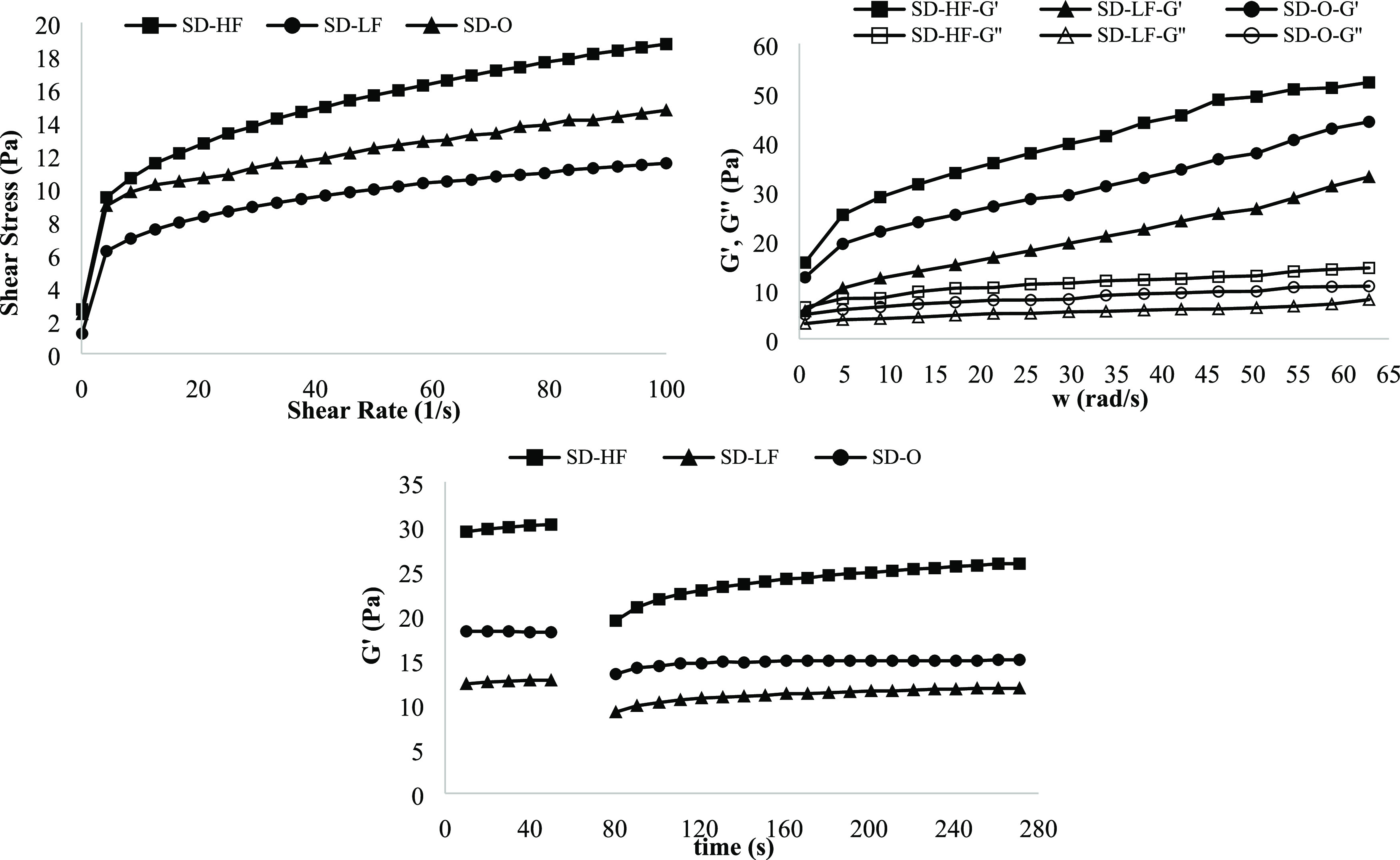
Rheological properties of the control and optimum salad dressing samples. SD-LF, low-fat control salad dressing sample; SD-HF, high-fat control salad dressing sample; SD-O, low-fat optimum salad dressing sample.

Figure 3 indicates the steady-shear, dynamic, and thixotropic properties. In Figure 3a, shear stress values of the samples were examined against the shear rate change. As the shear rate increases, the shear stress increases in the shear-thinning behavior, related to the increased alignment of the constituent molecules.^32^ In other words, salad dressing samples showed pseudoplastic flow characteristics. In Figure 3b, the response to a stress sweep indicating the linear viscoelastic region is defined by the storage modulus (*G*′) and the loss modulus (*G*″) as a function of frequency (0–62.5 rad s^–1^). The magnitude of both *G*′ and *G*″ increased with frequency. In Figure 3c, the change in the *G*′ value of the salad dressing samples is given over time. As can be seen in Figure 3, the degree of recovery of the sample as a result of a sudden deformation varies depending on the applied shear rate, in other words, the deformation value. It is observed that as the deformation value increases, the ability of each sample to recover itself decreases.

#### 3.3.2 Emulsion Stabilities of SD-O, SD-LF, and SD-HF

Salad dressing is an O/W emulsion with 30% oil content, and hence, it is thermodynamically unstable and always breaks down over time due to the unfavorable contact between the oil and water phases.^33,34^ The food industry prefers emulsifiers that have both hydrophilic and hydrophobic groups in their structure and stabilize the emulsion of two immiscible liquids to produce kinetically stable emulsions. The emulsifiers decrease the interfacial tension and hence play a significant role in the manufacture of very stable emulsions.^34^

**Figure 4:**
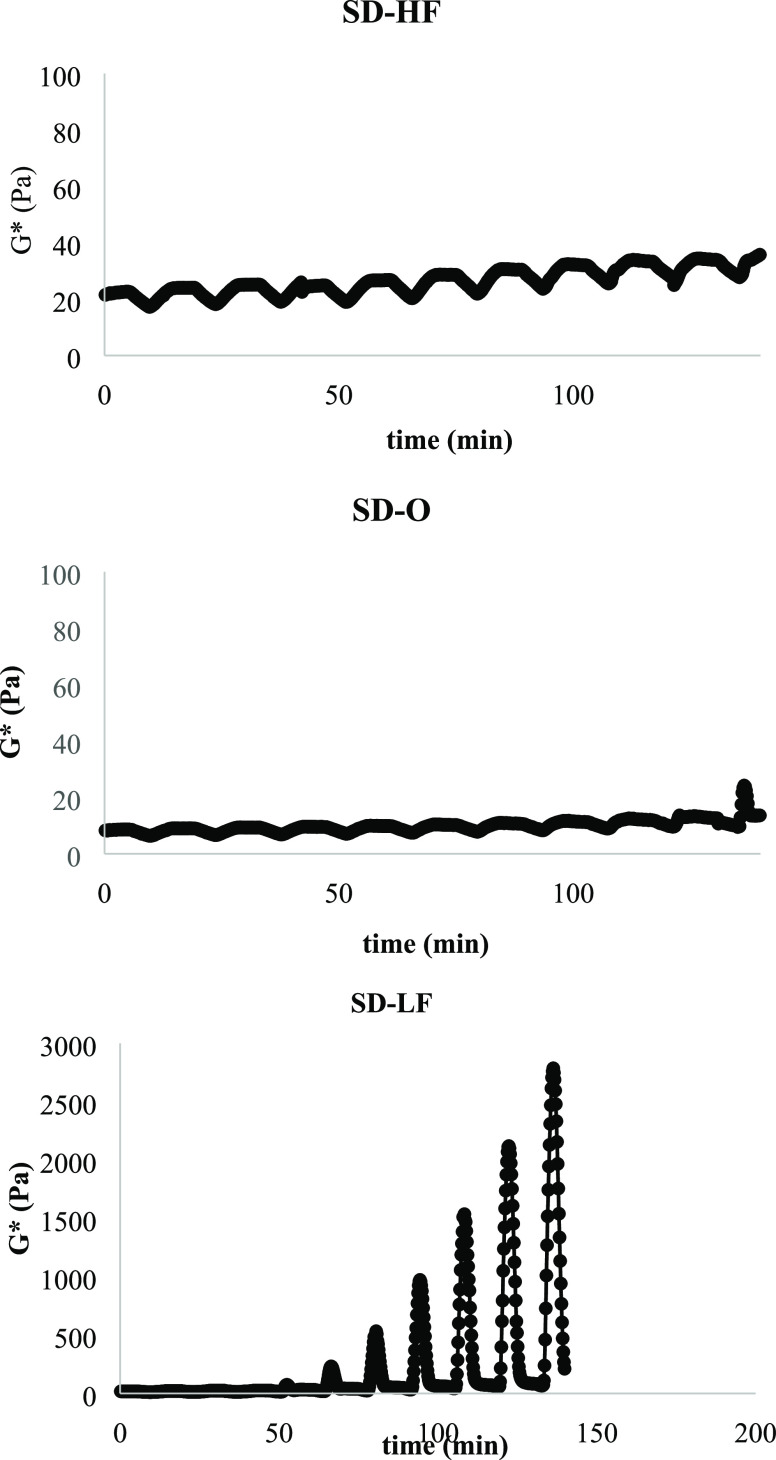
High-temperature thermal loop test of control and optimum salad dressing samples. SD-LF, low-fat control salad dressing sample; SD-HF, high-fat control salad dressing sample; SD-O, low-fat optimum salad dressing sample.

Emulsion stability is one of the most important quality parameters of salad dressing samples, as phase separation can be observed on the surface during storage in salad dressings with low emulsion stability. According to the study reported by Tekin, Avci, Karasu, and Toker,^20^ emulsion stability was determined by the thermal loop test based on the changes in G*, and higher changes in G* showed emulsion instability in thermally induced cycles. Figure 4 shows the changes in G* values in the high-temperature (25–45 °C) loop tests for salad dressing samples by applying 10 different loops. In Figure 4, the interaction of XG and TBP provided a solid structure and improved the physical stability of low-fat salad dressings. During the high-temperature thermal loop test in Figure 4, a dramatic change was observed in G* for the high-fat control sample following the low-fat control sample. As seen in Figure 4, the addition of 2.925% TBP to the low-fat mayonnaise sample provides physical stability.

#### 3.3.3 Oxidative Stabilities of SD-O, SD-LF, and SD-HF

The oxidative stabilities of SD-O, SD-LF, and SD-HF were determined by the OXITEST device at 80, 90, 100, and 110 °C, and the IP values of the samples are given in Table 6. IP values of SD-HF, SD-LF, and SD-O samples were determined as 1.01–12.82, 0.66–10.46, and 1.76–17.04 h, respectively. As can be seen, the SD-O sample enriched with TBP exhibited a significantly higher IP value. This result indicated that the tomato seed oil by-product could increase the oxidative stability of salad dressing samples. The low-fat sample showed the lowest IP value compared to the other samples. In oil/water emulsions, the oil fraction ratio is a vital parameter affecting oxidation stability. The oxidation stability decreases with the decrease in the oil fraction ratio in an emulsion.^35^ The significant increase in the IP value of the sample with by-product addition despite the decrease in oil can be explained by the scavenging of free radicals of the antioxidant components, especially the phenolic compounds in the by-product. In addition, the increase in the consistency value of the sample with by-product addition and thus the more compact dispersion of the oil droplets in the aqueous phase may also have caused an increase in the oxidative stability.

**Table 6:** Oxidation Kinetic Parameters of the Control and Optimum Salad Dressing Samples^a^^b^^c^

sample	temperature (°C)	IP (h)	*E*_a_(kJ mol^–1^)	Δ*H*^++^(kJ mol^–1^)	Δ*S*^++^(J mol^–1^ K^–1^)	Δ*G*^++^(kJ mol^–1^)
SD-HF	80	12.82^Ab^	97.168 ± 3.15^a^	94.113 ± 1.08^b^	20.04 ± 0.74^b^	86.437
	90	3.42^Bb^				86.638
	100	1.62^Cb^				86.838
	110	1.01^Db^				87.038
SD-LF	80	10.46^Ab^	104.674 ± 2.85^a^	101.619 ± 1.52^a^	42.6 ± 1.06^a^	85.303
	90	2.83^Bc^				85.729
	100	1.06^Cc^				86.155
	110	0.66^Dc^				86.581
SD-O	80	17.04^Aa^	88.516 ± 2.05^b^	85.861 ± 0.95^c^	–7.08 ± 0.47^c^	88.572
	90	7.13^Ba^				88.501
	100	2.52^Ca^				88.431
	110	1.76^Da^				88.360

a SD-LF, low-fat control salad dressing sample; SD-HF, high-fat control salad dressing sample; SD-O, low-fat optimum salad dressing sample.

b Different lowercase letters in the same column indicate statistical differences between samples subjected to a different temperature (*P* < 0.05).

c Different uppercase letters in some columns indicate statistical differences among samples HF-SD, LF-SD, and SD-O (*P* < 0.05).

Nonlinear modeling was applied to calculate the Arrhenius and activation of complex parameters (*E*_a_, Δ*H*^++^, Δ*S*^++^, and Δ*G*^++^ values). Table 6 exhibits *E*_a_, Δ*H*^++^, Δ*S*^++^, and Δ*G*^++^ values. *E*_a_ values represent the minimum energy value required to initiate oxidation, and they were found to be 97.168, 104.674, and 88.516 kJ mol^–1^ for SD-HF, SD-LF, and SD-O, respectively. The higher the *E*_a_ value, the higher the energy required for oxidation, that is, a higher oxidative stability. The addition of TBP significantly reduced the *E*_a_ values of the samples compared to SD-LF. It was reported that the antioxidant type differently affected the *E*_a_ of the oil oxidation.^36^ Therefore, the *E*_a_ value cannot be used alone in the assessment of oxidative stability. Δ*H*^++^, Δ*S*^++^, and ΔG^++^ values were used to evaluate the effect of temperature on the oxidation kinetics of salad dressing samples. Δ*H*^++^ values of SD-HF, SD-LF, and SD-O were determined to be 94.113, 101.619, and 88.516, respectively, while the Δ*S*^++^ values were found to be 20.04, 42.60, and −7.08, respectively. In the literature, Δ*H*^++^ and Δ*S*^++^ values were similarly reported for salad dressing and oil oxidation by Tekin-Cakmak, Atik, and Karasu,^8^ Ghoush, Samhouri, Al-Holy, and Herald,^31^ and Hashemi, Brewer, Safari, Nowroozi, Abadi Sherahi, Sadeghi, and Ghafoori.^37^

The reduction in Δ*S*^++^ with the addition of TBP can be explained by the reduction in the free radical concentration caused by the hydrogen donation of TBP antioxidants and the loss of rotational freedom in the transiently activated complex. The Δ*G*^++^ value was computed to determine the oxidation rate quantitatively. A slower rate of oxidation and a greater oxidation stability are indicated by the higher value of Δ*G*^++^. The values of Δ*G*^++^ were 86.43–87.03, 85.30–86.58, and 88.36–88.57 for SD-HF, SD-LF, and SD-O, respectively. The obtained Arrhenius parameters, IP values, and activation complicated parameters were all in agreement with one another. These results indicated that the oxidative stability of low-fat salad dressing samples could be improved with the addition of TBP as well as the improvement of rheological properties. The increase in oxidative stability by the addition of TBP can be explained by the free radical scavenging activity of the phenolic antioxidant of TBP, which is localized to the oil–water interface.

#### 3.3.4 Survival of *L. plantarum* ELB90 in Salad Dressing Samples

**Figure 5:**
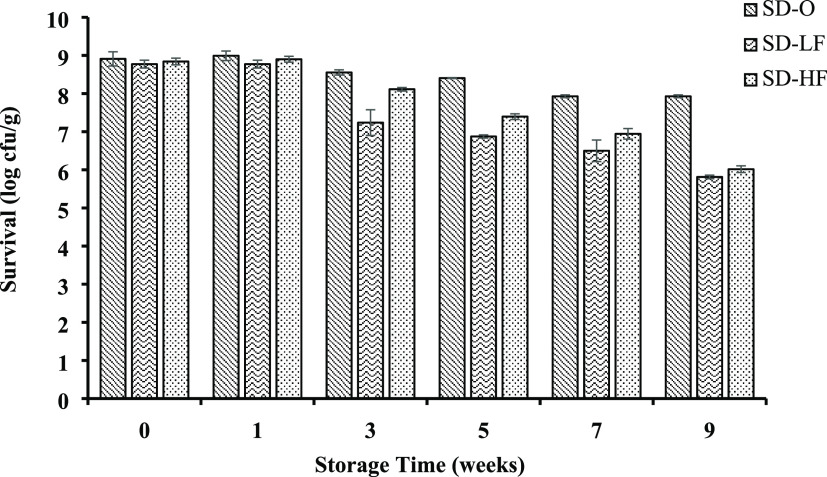
Viable *L. plantarum* ELB90 cell counts in salad dressing samples (SD-O, SD-LF, and SD-HF) during storage time.

The survival of probiotics until the end of storage at refrigerator temperature is the most important qualitative parameter for probiotic products. The initial counts of *L. plantarum* ELB90 in the optimum formulation, low-oil, and high-oil control salad dressings were 8.91, 8.77, and 8.84 log CFU g^–1^, respectively. Figure 5 shows the survival trends of *L. plantarum* ELB90 in the samples during the 9 week storage period under refrigerator temperatures.

At the end of storage, the *L. plantarum* count decreased by 2.96 log CFU g^–1^ for SD-LF and 2.82 log CFU g^–1^ for SD-HF samples, while a decrease of 0.98 log CFU g^–1^ was determined for the SD-O sample. It was observed that *L. plantarum* had better survivability in the SD-O formulation. Although the SD-O sample contained the same amount of oil as the SD-LF sample, the TBP content was highly effective in maintaining the viability of *L. plantarum*. There was no significant difference between samples in the count of *L. plantarum* at three weeks of storage. However, after 5 weeks of storage, the viability of *L. plantarum* in the SD-O sample was significantly higher, which persisted to the end of storage (*p* < 0.05). The food matrix including prebiotic ingredients stimulates the growth of probiotic bacteria. The proper combination of prebiotics and probiotics shows a higher potential for a synergistic effect.^38^ Defatted tomato seeds include a high level of insoluble dietary fiber (41.4%) and soluble dietary fiber (14.2%).^39^ Therefore, at the end of the storage, *L. plantarum* was counted as 7.93 log CFU g^–1^ in the SD-O formulation including TBP, while they were 5.81 log CFU g^–1^ in the SD-LF and 6.02 log CFU g^–1^ in SD-HF samples. Results indicated that the salad dressing including TBP could be a suitable vehicle for *L. plantarum* delivery.

## 4 Conclusions

The probiotic ability of *L. plantarum* to survive during long-term refrigerated storage, especially after 5 weeks of storage period, was improved by the addition of tomato seed oil by-product in a low-fat salad dressing formulation. In addition to enhancing the viability of *L. plantarum*, the presence of TBP in the low-fat salad dressing provided an appreciable improvement of its rheological properties and oxidative stability. Also, a functional low-fat salad dressing has been produced thanks to the physicochemical and bioactive properties of TBP in addition to its emulsion stabilization and thickening properties. As a conclusion, TBP may be an appropriate nondairy carrier for probiotics.

## References

[ref1] Mozafari H. R.; Hosseini E.; Hojjatoleslamy M.; Mohebbi G. H.; Jannati N. Optimization low-fat and low cholesterol mayonnaise production by central composite design. J. Food Sci. Technol. 2017, 54, 591–600. 10.1007/s13197-016-2436-0. 28298672 PMC5334215

[ref2] Ma Z.; Boye J. I. Advances in the Design and Production of Reduced-Fat and Reduced-Cholesterol Salad Dressing and Mayonnaise: A Review. Food Bioprocess Technol. 2013, 6, 648–670. 10.1007/s11947-012-1000-9.

[ref3] Yilmaz E.; Aydeniz B.; Güneşer O.; Arsunar E. S. Sensory and physico-chemical properties of cold press-produced tomato (*Lycopersicon esculentum* L.) seed oils. J. Am. Oil Chem. Soc. 2015, 92, 833–842. 10.1007/s11746-015-2648-x.

[ref4] Ayduğan A.; Ok S.; Yılmaz E. Cold-Pressed Milk Thistle Seed Oil: Physico-Chemical Properties, Composition and Sensory Analysis. Grasas y Aceites 2022, 73, e481.

[ref5] Srikaeo K.; Poungsampao P.; Phuong N. T. Utilization of the Fine Particles Obtained from Cold Pressed Vegetable Oils: A Case Study in Organic Rice Bran, Sunflower and Sesame Oils. J. Oleo Sci. 2017, 66, 21–29. 10.5650/jos.ess16131. 27928142

[ref6] Tekin-Cakmak Z. H.; Karasu S.; Kayacan-Cakmakoglu S.; Akman P. K. Investigation of potential use of by-products from cold-press industry as natural fat replacers and functional ingredients in a low-fat salad dressing. J. Food Process. Preserv. 2021, 45, e15388 10.1111/jfpp.15388.

[ref7] Tekin Z. H.; Karasu S. Cold-pressed flaxseed oil by-product as a new source of fat replacers in low-fat salad dressing formulation: steady, dynamic and 3-ITT rheological properties. J. Food Process. Preserv. 2020, 44, e14650 10.1111/jfpp.14650.

[ref8] Tekin-Cakmak Z. H.; Atik I.; Karasu S. The Potential Use of Cold-Pressed Pumpkin Seed Oil By-Products in a Low-Fat Salad Dressing: The Effect on Rheological, Microstructural, Recoverable Properties, and Emulsion and Oxidative Stability. Foods 2021, 10, 2759 10.3390/foods10112759. 34829043 PMC8620466

[ref9] Akcicek A.; Karasu S. Utilization of cold pressed chia seed oil waste in a low-fat salad dressing as natural fat replacer. J. Food Process Eng. 2018, 41, e12694 10.1111/jfpe.12694.

[ref10] Rigo-Adrover M. d. M.; Franch À.; Castell M.; Pérez-Cano F. J. Preclinical Immunomodulation by the Probiotic Bifidobacterium breve M-16V in Early Life. PLoS One 2016, 11, e0166082 10.1371/journal.pone.0166082. 27820846 PMC5098803

[ref11] Vijaya Kumar B.; Vijayendra S. V.; Reddy O. V. Trends in dairy and non-dairy probiotic products - a review. J. Food Sci. Technol. 2015, 52, 6112–6124. 10.1007/s13197-015-1795-2. 26396359 PMC4573104

[ref12] Mantzouridou F.; Karousioti A.; Kiosseoglou V. Formulation optimization of a potentially prebiotic low-in-oil oat-based salad dressing to improve *Lactobacillus paracasei* subsp. paracasei survival and physicochemical characteristics. LWT--Food Sci. Technol. 2013, 53, 560–568. 10.1016/j.lwt.2013.04.005.

[ref13] Mantzouridou F.; Spanou A.; Kiosseoglou V. An inulin-based dressing emulsion as a potential probiotic food carrier. Food Res. Int. 2012, 46, 260–269. 10.1016/j.foodres.2011.12.016.

[ref14] AOAC. Official Methods of Analysis of the Association of Official Analytical Chemistry AOAC — Association of Official Analytical Chemistry. American Association of Cereal Chemists. Approved Methods of the AACC, 10th Ed. Methods 30-25, 44-15A, 46-30. The Association: St. Paul, MN, 17th ed.; AOAC: Washington, 2002.

[ref15] Singleton V. L.; Orthofer R.; Lamuela-Raventós R. M. [14] Analysis of Total Phenols and Other Oxidation Substrates and Antioxidants by Means of Folin-Ciocalteu Reagent. In Methods in Enzymology; Academic Press, 1999; Vol. 299, pp 152–178.

[ref16] avon Gadow A.; Joubert E.; Hansmann C. F. Comparison of the antioxidant activity of aspalathin with that of other plant phenols of rooibos tea (*Aspalathus linearis*), α-tocopherol, BHT, and BHA. J. Agric. Food Chem. 1997, 45, 632–638. 10.1021/jf960281n.

[ref17] Tekin Z. H.; Karasu S. Cold-pressed flaxseed oil by-product as a new source of fat replacers in low-fat salad dressing formulation: Steady, dynamic and 3-ITT rheological properties. J. Food Process. Preserv. 2020, 44, e14650 10.1111/jfpp.14650.

[ref18] YOO B.; Rao M. Creep and dynamic rheological behavior of tomato concentrates: effect of concentration and finisher screen size. J. Texture Stud. 1996, 27, 451–459. 10.1111/j.1745-4603.1996.tb00087.x.

[ref19] Toker O. S.; Karasu S.; Yilmaz M. T.; Karaman S. Three interval thixotropy test (3ITT) in food applications: A novel technique to determine structural regeneration of mayonnaise under different shear conditions. Food Res. Int. 2015, 70, 125–133. 10.1016/j.foodres.2015.02.002.

[ref20] Tekin Z. H.; Avci E.; Karasu S.; Toker O. S. Rapid determination of emulsion stability by rheology-based thermal loop test. LWT 2020, 122, 109037 10.1016/j.lwt.2020.109037.

[ref21] Karasu S.; Toker O. S.; Yilmaz M. T.; Karaman S.; Dertli E. Thermal loop test to determine structural changes and thermal stability of creamed honey: Rheological characterization. J. Food Eng. 2015, 150, 90–98. 10.1016/j.jfoodeng.2014.10.004.

[ref22] Aksoy F. S.; Tekin-Cakmak Z. H.; Karasu S.; Aksoy A. S. Oxidative stability of the salad dressing enriched by microencapsulated phenolic extracts from cold-pressed grape and pomegranate seed oil by-products evaluated using OXITEST. Food Sci. Technol. 2022, 42, e57220 10.1590/fst.57220.

[ref23] Yildirim R. M.; Arici M. Effect of the fermentation temperature on the degradation of phytic acid in whole-wheat sourdough bread. LWT 2019, 112, 108224 10.1016/j.lwt.2019.05.122.

[ref24] Karaman S.; Karasu S.; Tornuk F.; Toker O. S.; Geçgel Ü.; Sagdic O.; Ozcan N.; Gül O. Recovery Potential of Cold Press Byproducts Obtained from the Edible Oil Industry: Physicochemical, Bioactive, and Antimicrobial Properties. J. Agric. Food Chem. 2015, 63, 2305–2313. 10.1021/jf504390t. 25647068

[ref25] Konuşkan D. B. Minor Bioactive Lipids in Cold Pressed Oils. In Cold Pressed Oils; Elsevier, 2020; pp 7–14.

[ref26] Batista A. P.; Raymundo A.; Sousa I.; Empis J.; Franco J. M. Colored food emulsions—Implications of pigment addition on the rheological behavior and microstructure. Food Biophys. 2006, 1, 216–227. 10.1007/s11483-006-9022-3.

[ref27] Diftis N.; Biliaderis C.; Kiosseoglou V. Rheological properties and stability of model salad dressing emulsions prepared with a dry-heated soybean protein isolate–dextran mixture. Food Hydrocolloids 2005, 19, 1025–1031. 10.1016/j.foodhyd.2005.01.003.

[ref28] Ma L.; Barbosa-Cánovas G. Rheological characterization of mayonnaise. Part II: Flow and viscoelastic properties at different oil and xanthan gum concentrations. J. Food Eng. 1995, 25, 409–425. 10.1016/0260-8774(94)00010-7.

[ref29] Puppo M. C.; Sorgentini D.; Anon M. Rheological study of dispersions prepared with modified soybean protein isolates. J. Am. Oil Chem. Soc. 2000, 77, 63–71. 10.1007/s11746-000-0010-z.

[ref30] Raymundo A.; Franco J.; Empis J.; Sousa I. Optimization of the composition of low-fat oil-in-water emulsions stabilized by white lupin protein. J. Am. Oil Chem. Soc. 2002, 79, 783–790. 10.1007/s11746-002-0559-6.

[ref31] Ghoush M. A.; Samhouri M.; Al-Holy M.; Herald T. Formulation and fuzzy modeling of emulsion stability and viscosity of a gum–protein emulsifier in a model mayonnaise system. J. Food Eng. 2008, 84, 348–357. 10.1016/j.jfoodeng.2007.05.025.

[ref32] Rha C. Theories and Principles of Viscosity. In Theory, Determination and Control of Physical Properties of Food Materials; Springer, 1975; pp 7–24.

[ref33] Depree J.; Savage G. Physical and flavour stability of mayonnaise. Trends Food Sci. Technol. 2001, 12, 157–163. 10.1016/S0924-2244(01)00079-6.

[ref34] McClements D. J.; Weiss J.; Corradini M. G. Lipid emulsions. Bailey’s Ind. Oil Fat Prod. 2005, 1–40. 10.1002/047167849X.bio019.pub2.

[ref35] Berton-Carabin C. C.; Ropers M.-H.; Genot C. Lipid Oxidation in Oil-in-Water Emulsions: Involvement of the Interfacial Layer. Compr. Rev. Food Sci. Food Saf. 2014, 13, 945–977. 10.1111/1541-4337.12097.

[ref36] Farhoosh R. A Kinetic Approach to Evaluate the Structure-Based Performance of Antioxidants During Lipid Oxidation. J. Food Sci. 2018, 83, 101–107. 10.1111/1750-3841.13993. 29210460

[ref37] Hashemi S. M. B.; Brewer M. S.; Safari J.; Nowroozi M.; Abadi Sherahi M. H.; Sadeghi B.; Ghafoori M. Antioxidant Activity, Reaction Mechanisms, and Kinetics of Matricaria recutita Extract in Commercial Blended Oil Oxidation. Int. J. Food Prop. 2016, 19, 257–271. 10.1080/10942912.2015.1020438.

[ref38] Ranadheera R. D. C. S.; Baines S. K.; Adams M. C. Importance of food in probiotic efficacy. Food Res. Int. 2010, 43, 1–7. 10.1016/j.foodres.2009.09.009.

[ref39] Kumar M.; Chandran D.; Tomar M.; Bhuyan D. J.; Grasso S.; Sá A. G. A.; Carciofi B. A. M.; Radha; Dhumal S.; Singh S.; et al. Valorization Potential of Tomato (*Solanum lycopersicum* L.) Seed: Nutraceutical Quality, Food Properties, Safety Aspects, and Application as a Health-Promoting Ingredient in Foods. Horticulturae 2022, 8, 265 10.3390/horticulturae8030265.

